# Profil de l'infection urinaire nosocomiale dans un service de nephrology

**DOI:** 10.11604/pamj.2014.19.59.4835

**Published:** 2014-09-23

**Authors:** Mohamed Amine Lazrak, Ghita El Bardai, Soumia Jaafour, Nadia Kabbali, Mohamed Arrayhani, Tarik Sqalli Houssaini

**Affiliations:** 1 Service de Néphrologie, CHU Hassan II de Fès, Maroc; 2 Faculté de Médecine de Fès, Maroc

**Keywords:** Infection urinaire, nosocomiale, facteurs de risque, néphrologie, urinary infection, hospital acquired, risk factor, nephrology

## Abstract

L'infection urinaire est l'infection nosocomiale la plus fréquente. Elle constitue un véritable problème de santé publique par la surmortalité et le surcoût qu'elle entraîne. L'objectif de notre étude est de déterminer l'incidence et le profil des IU nosocomiales dans un service de Néphrologie. Etude rétrospective sur dossier de tous les patients hospitalisés dans notre service durant l'année 2011, ayant bénéficié d'un examen cytobactériologique des urines. Ont été exclus, tous les patients admis avec une IU connue ou active. 325 dossiers ont été retenus. L'incidence de l'IU nosocomiale était de l'ordre de 16,9%. La durée moyenne d'hospitalisation était de 14,1±10,15 jours. 30% de nos patients ont été transférés du service des urgences. 80% des IU nosocomiales étaient compliquées. Le germe responsable était E.Coli dans 2/3 des cas dont 14,5% était à E.Coli sécrétrice de bétalactamases à spectre étendu. L’évolution après traitement était favorable chez 90,7%. En analyse multivariée, les facteurs de risque pour contracter une IU nosocomiale étaient le sexe féminin; le sondage urinaire et l'antécédent d'IU à répétition. Nos résultats rejoignent ceux de la littérature concernant les facteurs de risque liés à la survenue de l'IU nosocomiale, la fréquence des infections à entérobactéries, et l’émergence de souches résistantes. Une Surveillance microbiologique et une évaluation de la résistance aux antibiotiques constituent une ligne de défense pour faire face à l'accentuation de nouvelles souches bactériennes de plus en plus résistantes aux antibiotiques rendant les options thérapeutiques très limitées.

## Introduction

L'infection urinaire est l'infection nosocomiale la plus fréquente. Elle représente jusqu′à 40% de toutes les infections acquises à l'hôpital [1]. Elle occupe une place importante en pathologie néphrologique, et constitue un véritable problème de santé publique par la surmortalité et le surcoût qu'elle entraîne. Les patients porteurs de sondes urinaires, ou ayant un séjour de longue durée et les patients âgés atteints de maladies débilitantes sont à risque élevé de développer des infections urinaires nosocomiales(IUN). Les organismes responsables proviennent généralement de la flore intestinale endogène des patients, ou à l′occasion d′un contact avec un site infecté dans le milieu hospitalier. Le meilleur traitement reste la prévention. L'objectif de cette étude est de déterminer la prévalence des IUN dans le service de Néphrologie du Centre Hospitalier Universitaire Hassan II de Fès (Maroc), leur caractère simple ou compliqué, et de rechercher les facteurs de risque liés à leur survenue.

## Méthodes

**Population de l’étude:** Il s'agit d'une étude rétrospective incluant tous les patients hospitalisés dans le service de néphrologie du Centre Hospitalier Universitaire Hassan II de Fès durant la période allant du 1er janvier au 31 décembre 2011, ayant tous bénéficié d'un examen cytobactériologique des urines (ECBU). Ont été exclus, tous les patients admis avec une infection urinaire connue ou active.

**Définitions et méthodologie:** Une infection est dite nosocomiale dans les cas suivants [2]: Cas 1: Si aucune infection antérieure de l'arbre urinaire n’était présente ou en incubation à l'admission. Cas 2: Si une infection antérieure de l'arbre urinaire était présente mais le microorganisme identifié est différent, ou l'infection précédente était déclarée comme guérie. Cas 3: Si l’état à l'admission n'est pas connu et l'infection est apparu après un délai de 48 heures. L'infection urinaire nosocomiale (IUN) est définie par la présence d'au moins un des signes suivants [3]: Fièvre (> 38°C), impériosité mictionnelle, pollakiurie, brulure mictionnelle, douleur sus-pubienne en l'absence d'autre cause, infectieuse ou non. Et: Sans sondage vésical ni autre abord de l'arbre urinaire: (Leucocyturie ≥ 10^4^ leucocytes/ml) et uroculture positive ( ≥10^3^ microorganisme/ml) et au plus deux microorganismes différents avec sondage vésical et autre abord de l'arbre urinaire, en cours ou dans les sept jours précédents: uroculture positive (≥ 10^5^ microorganismes /ml) et au plus deux microorganismes différents. Une infection urinaire est dite compliquée en présence d'une pathologie organique ou fonctionnelle (reflux vésico-urétéral; lithiase; tumeur), une situation pathologique (diabète; insuffisance rénale; immunodépression) ou un terrain physiologique particulier (le sexe masculin; la grossesse; un sujet âgé ayant une comorbidité) [4]. Nous avons recueillis et analysé les données suivantes: Paramètres cliniques: Age, sexe, antécédents de diabète, d'infection urinaire à répétition de lithiase et d’énurésie; Paramètres liées à l'infection urinaire: Fièvre, impériosité mictionnelle, pollakiurie, brulure mictionnelle, sondage urinaire; Paramètres biologiques: ECBU, Antibiogramme, Fonction rénale, CRP, Numération Formule Sanguine; Paramètres radiologiques: données de l’échographie rénale, réalisée chez toute la population de l’étude.

**Analyse statistique:** Au plan statistique, les données ont été saisies sur Microsoft office Excel 2010. L'analyse statistique a été effectuée en utilisantlogiciel SPSS 17.0, en collaboration avec le laboratoire d’épidémiologie et de recherche clinique de la Faculté de Médecine de Fès. Les variables quantitatives ont été exprimées en moyenne et les variables qualitatives en pourcentage. La comparaison des variables qualitatives a été effectuée par le test Khi-deux de Pearson et le test exact de Fisher. Une valeur de p < 0,05 a été considérée comme significative. En analyse multivariée, un modèle de régression logistique a été réalisé en utilisant la procédure de pas à pas descendants.

## Résultats

**Figure 1:**
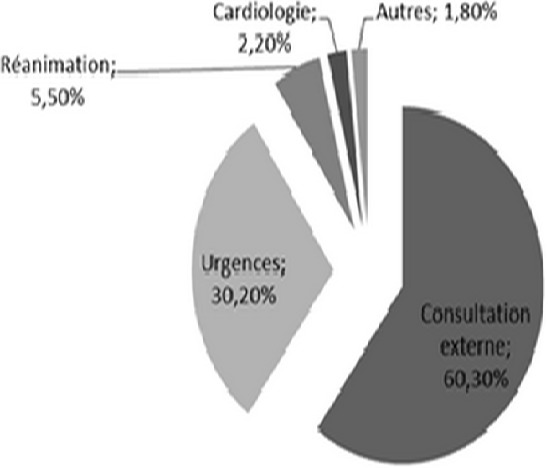
Répartition selon les services de provenance

**Figure 2:**
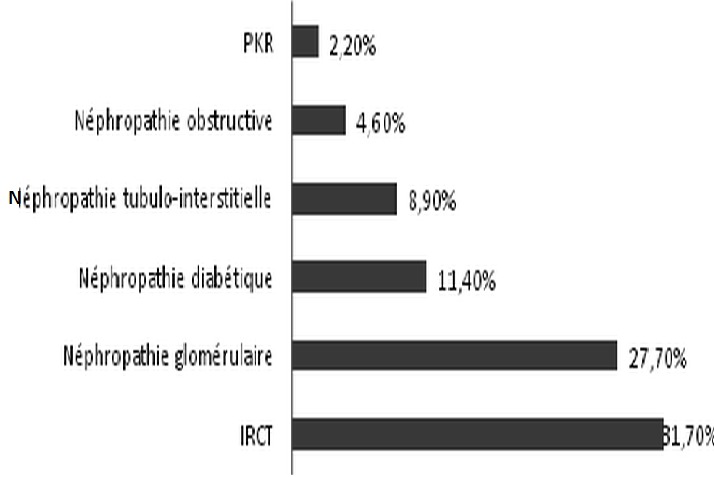
Les motifs d'hospitalisation chez nos patients

**Figure 3:**
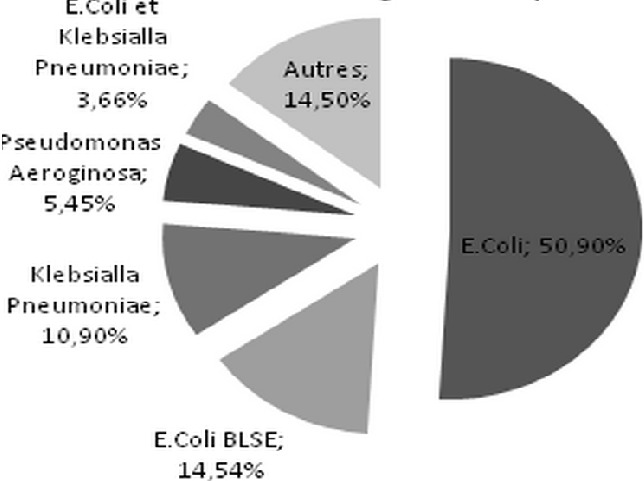
Fréquence des germes isolés sur les ECBU des patients ayant une IUN

Durant la période de l’étude, 325 dossiers ont été retenus répondant aux critères d'inclusion, 9,41% des patients hospitalisés présentant une infection urinaire connue ou active à leur admission. L’âge moyen de nos patients était de 51± 18 ans, avec un sex-ratio à 1. La durée moyenne de séjour était de 14,1±10 jours. 60,3% de nos malades ont été recrutés de la consultation externe, tandis que 30% ont été transférés du service des urgences (Figure 1). Un tiers des patients avaient une insuffisance rénale chronique, 27,7% avait une néphropathie glomérulaire et 2,2% étaient porteurs d'une polykystose rénale (Figure 2). L’échographie rénale était normale chez 59,1% de nos patients. Presque un quart des patients avaient des reins de petite taille. Seulement 4,3% avaient une dilatation des cavités pyélo-calicielles et 3,4% étaient porteurs d'un ou de plusieurs kystes rénaux. Un sondage urinaire a été réalisé dans 10,2% des cas. La prévalence de l'IUN dans notre étude était de l'ordre de 16,9%. Elle était compliquée dans 80% des cas. Le germe responsable était un Escherichia coli (E.Coli) dans les 2/3 des cas, dont 14,5% était à E.Coli à Bêtalactamases à spectre étendu (BLSE) et une Klebsiella Pneumoniae dans 11% des cas (Figure 3).

L’évolution après traitement était favorable chez 90,7% des patients et marquée par la survenue d'une septicémie à point de départ urinaire dans 2% des cas, 4% de nos patients ont eu une réinfection et 3,2% une rechute. En analyse univariée, l’âge, le sexe féminin, le sondage urinaire, la lithiase urinaire, le passage par le service des urgences, les antécédents d'infections urinaires à répétition, et la durée prolongée d'hospitalisation étaient des facteurs de risque de survenue de l'IUN (Tableau 1). En analyse multivariée par méthode de régression logistique pas à pas descendant, les facteurs de risque pour contracter une IUN étaient: le sexe féminin (OR = 6,7; IC à 95%: 2,6-17,0); le sondage urinaire (OR = 33,6; IC à 95%: 11,5-98,0) et l'infection urinaire à répétition (OR = 11,8; IC à 95%:3,2-43,4) (Tableau 2).

**Tableau 1:** Facteurs de risque des IUN identifiés après analyse univariée

Paramètres	Infection urinaire		p
	absente	présente	
Age (ans)	49,6 ± 18	57,7 ± 16,5	0,003
Sexe ratio	119 F/151 H	41 F / 14 M	0,0001
Sonde urinaire (%)	3,33	43,6	0,0001
Diabète (%)	16,3	14,5	NS
Chirurgie (%)	1,85	7,27	0,04
Lithiase (%)	5,18	12,7	0,06
Infection urinaire à répétition (%)	1,48	23,6	0,000
Provenance des urgences (%)	27,4	43,6	0,001
Durée de séjour hospitalier (jours)	13,6 ± 10	16,6 ± 7,8	0,04

**Tableau 2:** Facteurs de risque des IUN identifiés en analyse multivariée

	A	E.S.	Wald	ddl	Sig.	Exp(B)	IC pour Exp(B) 95%	
							Inférieur	Supérieur
Sexe féminin	1,899	0,477	15,874	1	0,000	6,680	2,625	17,004
Sondage urinaire	3,516	0,545	41,586	1	0,000	33,662	11,561	98,013
IU à répétition	2,469	0,664	13,816	1	0,000	11,814	3,213	43,438
Constante	-3,606	0,449	64,531	1	0,000	0,027		

## Discussion

L'infection urinaire constitue l'infection nosocomiale la plus fréquente puisqu'elle représente presque la moitié de l'ensemble des infections nosocomiales [5], surtout dans les unités de soins intensifs, les services d'urologie et de néphrologie en vue de la fréquence élevée des sondages urinaires. La prévalence des IUN dans notre série était de l'ordre de 16,9%, elle reste cependant plus élevée par rapport à celles rapportées dans la littérature, notamment dans une série canadienne où la prévalence des IUN était de 9,5% [6]. Cette prévalence élevée d'IUN dans notre série pourrait être expliquée par le recrutement fréquent de patients ayant séjourné au service des urgences (30% environ) et ayant été sujets à des sondages urinaires itératifs, ou bien par la réalisation d'un ECBU de façon systématique au cours de leurs hospitalisation dans le service de néphrologie, ainsi qu'une durée moyenne de séjour hospitalier qui reste relativement prolongée (14,1±10 jours).

Pour ce qui est des germes isolés, les bacilles Gram négatifs sont les plus fréquemment identifiés dans les milieux de culture au cours de notre étude, et en chef de fil l'E. Coli, la Klebsiella Pneumoniae et le Pseudomonas Aeroginosa. Ceci a également été retrouvé dans plusieurs séries internationales [7–9]. De récentes données américaines indiquent qu'E. Coli est étiologiquement le plus commun des micro-organismes Gram négatifs isolés lors des IUN, suivie dans un ordre décroissant de fréquence, par Pseudomonas Aeroginosa, Klebsiella Pneumoniae, Enterobacter, et Acinétobacter Baumannii [10]. Les souches d'E. Coliuropathogènes infectent le tractus urinaire à travers une série de mécanismes comme les adhésines spécialisés, le biofilm, et l′aversion de l′hôte. L′émergence de la résistance aux quinolones et aux céphalosporines à spectre étendu demeure un défi considérable, car ces agents sont souvent utilisés comme traitement de première intention [11]. Dans notre série, l'E.Coli était responsable dans les 2/3 des cas d'IUN dont 22,2% de souche BLSE, ce qui est en similitude avec une série turque où l'E.Coli a été incriminé dans 40,8% des IUN dont 27% étaient sécrétrices de BLSE [12].

Beaucoup d’études internationales ont identifié plusieurs facteurs de risque pour développer une colonisation ou une infection parmicro-organismes producteurs de BLSE comme [13–15]: -Durée du séjour hospitalier; -Longueur du séjour en réanimation; Présence de cathéter veineux central; -La chirurgie abdominale d′urgence et la présence d′une gastrostomie ou jéjunostomie; -L′administration préalable d′un antibiotique; -Gravité de la maladiesous-jacente; -Présence d′une sonde urinaire; -L'assistance ventilatoire; -Hémodialyse. Il a été démontré que le tube digestif constitue le principal réservoir d'entérobactéries productrices de BLSE [16]. Un voyage vers l′Asie semble également être un facteur de risque; la gastro-entérite contractée au cours d′un voyage peut être un paramètre de substitution pour ce qui est du contact avec l′eau ou des aliments contaminés par des matières fécales. Cela a été illustré dans une étude prospective incluant 100 adultes suédois qui,deretour chez eux après une durée moyenne de séjour de 02 semaines, étaient colonisés par une souche E. coli productrices de BLSE dans presque le ¼ des cas^17^. Les facteurs de risque liés à la survenue d'IUN dans notre étuderejoignent ceux de la littérature, à savoir: le sexe féminin, le sondage urinaire et l'infection urinaire à répétition [7, 18]. Dans notre série le diabète et la lithiase urinaire ne sont pas ressortis comme facteurs de risque statistiquement significatifs, ceci est expliqué du fait que le pourcentage des patients connus diabétiques ou porteurs de lithiase urinaire est presque à pied d’égalité dans les deux groupes comparés (groupes porteurs d'IUN et groupe non infecté). Une étude Sud-Coréenne récemment publiée [19] suggère la limitation de l′utilisation des fluoroquinolones et la réduction des procédures invasives telles que le sondage urinaire comme moyen de contrôle sur les facteurs de risque favorisant la multirésistance de la bactériémie nosocomiale à E.Coli et à Klebsiella Pneumoniae.

Les politiques de contrôle des infections nosocomiales à travers des commissions ou des groupes de travail au sein de l'hôpital sont importantes à instaurer afin de limiter le nombre d′IUN. D′autres points importants comprennent l’éviction du sondage urinaire itératif ainsi que le respect strict des règles universelles d'hygiène lors du cathétérisme urinaire en utilisant une technique aseptique, un matériel stérile et des systèmes de drainage fermés. Limiter la durée d'hospitalisation permettrait aussi de réduire le risque de survenue d'une IUN et d’économiser en plus des dépenses évitables pour l'hôpital. La place des cathéters imprégnés d′antibiotiques reste à préciser, mais leur utilisation pourrait conduire également à une réduction de l′incidence des IUN [19].

## Conclusion

L'infection nosocomiale contribue à la morbidité et la mortalité des patients à l'hôpital. La fréquence élevée de l'IUN dans notre série pourrait être expliquée par le passage au service des urgences ainsi que le sondage urinaire excessif, ceci impose l’éviction du sondage urinaire non justifié, ainsi que l'isolement des patients infectés au sein des services hospitaliers. Une Surveillance microbiologique en temps opportun et une évaluation de la résistance aux antibiotiques constituent une ligne de défense pour faire face à l’émergence de nouvelles souches bactériennes de plus en plus résistantes aux antimicrobiens à large spectre rendant les options thérapeutiques de en plus limitées.
